# Early dexamethasone treatment for septic shock patients: a prospective randomized clinical trial

**DOI:** 10.1590/S1516-31802007000400009

**Published:** 2007-07-01

**Authors:** Domingos Dias Cicarelli, Joaquim Edson Vieira, Fábio Ely Martins Benseñor

**Keywords:** Infection, Septic shock, Sepsis, Glucocorticoids, Dexamethasone, Infecção, Choque séptico, Sepse, Glucocorticóides, Dexametasona

## Abstract

**CONTEXT AND OBJECTIVE:**

Sepsis and septic shock are very common conditions among critically ill patients that lead to multiple organ dysfunction syndrome (MODS) and death. Our purpose was to investigate the efficacy of early administration of dexamethasone for patients with septic shock, with the aim of halting the progression towards MODS and death.

**DESIGN AND SETTING:**

Prospective, randomized, double-blind, single-center study, developed in a surgical intensive care unit at Hospital das Clínicas, Faculdade de Medicina da Universidade de São Paulo.

**METHODS:**

The study involved 29 patients with septic shock. All eligible patients were prospectively randomized to receive either a dose of 0.2 mg/kg of dexamethasone (group D) or placebo (group P), given three times at intervals of 36 hours. The patients were monitored over a seven-day period by means of the sequential organ failure assessment score.

**RESULTS:**

Patients treated with dexamethasone did not require vasopressor therapy for as much time over the seven-day period as did the placebo group (p = 0.043). Seven-day mortality was 67% in group P (10 out of 15) and 21% in group D (3 out of 14) (relative risk = 0.31, 95% confidence interval 0.11 to 0.88). Dexamethasone enhanced the effects of vasopressor drugs.

**CONCLUSIONS:**

Early treatment with dexamethasone reduced the seven-day mortality among septic shock patients and showed a trend towards reduction of 28-day mortality.

## RESUMO

**CONTEXTO E OBJETIVO:**

Sepse e choque séptico são doenças muito comuns em pacientes gravemente enfermos, evoluindo muitas vezes com síndrome de disfunção de múltiplos órgãos (SDMO) e morte. A proposta do trabalho foi investigar a eficácia da administração precoce de dexametasona a estes pacientes, tentando evitar a progressão do choque séptico para SDMO e morte.

**TIPO DE ESTUDO E LOCAL:**

Estudo prospectivo, aleatório, duplamente encoberto, monocêntrico, realizado na Unidade de Terapia Intensiva pós-operatória do Hospital das Clínicas da Faculdade de Medicina da Universidade de São Paulo.

**MÉTODOS:**

Foram estudados 29 pacientes com choque séptico. Os participantes foram aleatoriamente divididos em dois grupos que receberam 0,2 mg/kg de dexametasona (grupo D) ou placebo (grupo P), repetidas a cada 36 horas. Os pacientes foram acompanhados durante sete dias de internação na Unidade de Terapia Intensiva através do escore SOFA (Sequential Organ Failure Assessment).

**RESULTADOS:**

Os pacientes que receberam dexametasona necessitaram de menos tempo de tratamento com vasopressores durante o período de sete dias (p = 0,043). A mortalidade em sete dias no grupo P foi de 67% (10 em 15) e no grupo D foi de 21% (3 em 14) (risco relativo = 0.31, intervalo de confiança 95% 0.11-0.88).

**CONCLUSÃO:**

O tratamento precoce com dexametasona dos pacientes com choque séptico reduziu a mortalidade em sete dias de acompanhamento e mostrou tendência de redução da mortalidade em 28 dias.

## INTRODUCTION

Septic shock results when infectious or inflammatory agent-induced mediators produce hemodynamic decompensation. Septic shock is defined as severe sepsis with hypotension despite adequate fluid resuscitation that requires vasopressor support. About half of the patients with septic shock die of multiple organ system failure. Multiple organ dysfunction syndrome (MODS) is defined as organ dysfunction in critically ill patients who require intervention to reach homeostasis maintenance.^1^

Glucocorticoids have an important immunosuppressive effect, reducing the transcription of proinflammatory genes by inhibition of the nuclear factor kappa B.^2,3^ Several studies have involved the use of corticosteroids to reduce the systemic inflammatory process associated with the host response to sepsis and septic shock.^4^ Several reports have been published recently on studies involving lower doses of hydrocortisone, which showed improved outcomes for patients suffering from septic shock. The use of methylprednisolone to obtain resolution of acute respiratory distress syndrome (ARDS) has also been studied.^5^

Currently, the recommendations for using corticosteroids to treat sepsis are that this class of drugs should be used during refractory septic shock, but not during severe sepsis in the absence of shock or when only mild shock is observed.^6^ Nonetheless, it needs to be asked why corticosteroids should not be used for septic patients at an early stage, before they evolve to refractory shock.

In a previous study,^7^ we used dexamethasone to treat systemic inflammatory response syndrome (SIRS) patients. We observed that a single dose of dexamethasone enhanced the effects of vasopressor drugs for an apparently temporary period, and that the respiratory system also presented improvements. Despite other recent studies ^4^ in which patients with septic shock were successfully treated with hydrocortisone, our previous study ^7^ revealed some advantages in using dexamethasone. This drug was chosen because of its potency and long-lasting action (36-48 hours) and its higher anti-inflammatory and lower mineralocorticoidal effects. In comparison with hydrocortisone, dexamethasone causes no changes in sodium reabsorption and does not interfere in the water balance, thus avoiding hypervolemia and sodium disturbances.^8^

## OBJECTIVE

This study aimed to evaluate the benefits from early administration of dexamethasone in patients with septic shock.

## METHODS

This study was prospective, randomized, double-blind and placebo-controlled. After approval by a local ethics committee, informed consent was obtained from patients or from their next of kin prior to enrollment.^9^ Twenty-nine patients admitted into the surgical intensive care unit of Hospital das Clínicas, Faculdade de Medicina da Universidade de São Paulo (HC/FMUSP) between November 2004 and December 2005 took part in the study. Three patients were excluded after their next of kin withdrew their consent.

Patients with septic shock diagnosed after admission into the intensive care unit (ICU) were eligible for the study. Patients aged under 18 years, patients with a history of immunosuppression therapy or a history of glucocorticoid use for over two weeks within the last year or upon admission to this hospital, and patients with active pancreatitis, terminal illness (end-stage neoplasm with a life expectancy of less than three months) or recent gastrointestinal hemorrhage were excluded.^5^

A randomization table determined the order of inclusion for the patients to receive placebo among the expected 30 admissions. All the eligible patients were prospectively randomized into two groups: Group D comprising 14 patients and Group P with 15 patients. Group D patients were given intravenous dexamethasone (0.2 mg/kg, three doses at intervals of 36 hours) while Group P patients received placebo (physiological saline solution 0.9%; three doses at intervals of 36 hours).^10^

The baseline severity of illness was assessed using the Acute Physiology and Chronic Health Evaluation II Score (APACHE II).^10^ Patients were assessed daily for seven consecutive days using the sequential organ failure assessment score (SOFA),^11-13^ or until their discharge from the ICU. Lactate plasma concentrations were also measured daily.^14^

The patients received conventional therapy with regard to antibiotic regimens, serial blood cultures (whenever their body temperature was greater than 38 º C) and discharge criteria. Relevant clinical and laboratory tests were conducted daily throughout the study. The subjects were evaluated during their stay in the ICU in relation to the duration of vasopressor support (SOFA score for cardiovascular system of two or more), duration of mechanical ventilation and mortality.

All patients who progressed to refractory septic shock, despite using high doses of norepinephrine (> 0.5 µg/kg/minute) and dobutamine (≥ 20 µg/kg/minute), were excluded from the study and administration of hydrocortisone (100 mg every 8 hours) was started.^6,15^

Statistical analysis was performed using the Sigma Stat for Windows program, version 2.03 (Statistical Package for the Social Sciences, SPSS Inc.). For continuous variables, the treatments were compared using the Student t test, Mann-Whitney U test and two-way analysis of variance (ANOVA) for the treatment and outcome conditions. Relative risk and confidence intervals were calculated for treated patients in relation to seven-day and 28-day mortality.^16^

## RESULTS

The mean age (± standard deviation, SD) of the 29 patients was 64 ± 13 years (range: 34 to 88 years). The study involved 13 males and 16 females (45%/55%). The mean age (± SD) of Group D was 69 ± 11 years while for Group P it was 61 ± 15 years (p = 0.12). There was no difference between these groups with regard to APACHE II (20 ± 5 for Group D and 19 ± 4 for Group P; p = 0.53). The baseline demographic characteristics and disease severity were similar in the placebo and dexamethasone groups (Table 1).

**Table 1.** Baseline characteristics of the patients with septic shock studied

Characteristics	Group P (n = 15)	Group D (n = 14)	p
Age (years)	61 ± 15	69 ± 11	0.12
Male Sex (%)	46.7	42.9	0.59
Weight (kg)	63.5 ± 11.7	68.5 ± 15.0	0.32
APACHE II score	19 ± 4	20 ± 5	0.53
SOFA score	10 ± 2	9 ± 3	0.44
**Prior or preexisting conditions (%)**				
Hypertension	28.6	33.3	
Myocardial infarction	14.3	13.3	
Diabetes	14.3	13.3	
Liver disease	7.1	-	
COPD	7.1	6.7	
Cancer	21.4	20	
Recent trauma	35.7	20	
**Other indicators of disease severity**				
Mechanical ventilation (days)	4.0 ± 3.2	3.4 ± 2.5	0.22
Shock (days of vasopressor use)	4.2 ± 1.9	3.4 ± 2.1	0.04

Group P = placebo; group D = dexamethasone; APACHE = Acute Physiology and Chronic Health Evaluation; SOFA = sequential organ failure assessment; COPD = chronic obstructive pulmonary disease.

**Figure 1.**
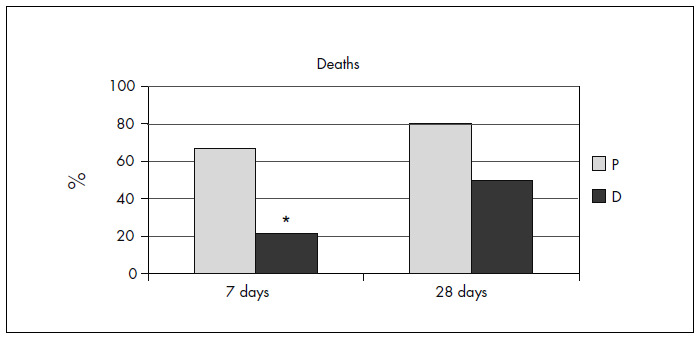
Comparison of mortality in Group D and Group P, for seven-day period (*relative risk, RR = 0.31; 95% confidence interval, CI: 0.11-0.88) and 28-day period (RR = 0.63; 95% CI: 0.31-1.29).

The seven-day mortality in Group P was 67% (10 out of 15) and in Group D it was 21% (3 out of 14) (relative risk = 0.31; 95% confidence interval: 0.11 to 0.88); the number needed to treat (NNT) was 2.17. The 28-day mortality in group P was 80% (12 out of 15) and in group D it was 50% (7 out of 14) (relative risk = 0.63; 95% confidence interval: 0.31 to 1.29) (Figure 1). With regard to collateral effects from dexamethasone (increased glucose, secondary infections or gastrointestinal hemorrhage), only one patient in Group P developed pneumonia (on the fourth postoperative day following aneurysm repair).

**Figure 2.**
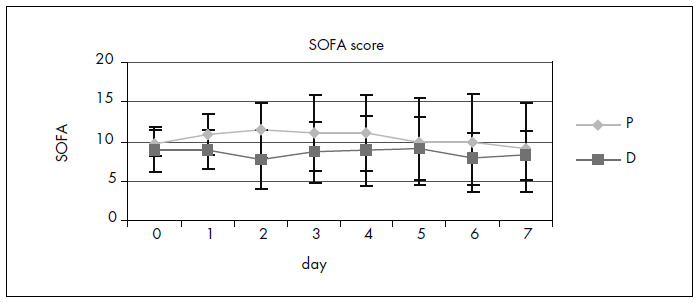
Evolution of sequential organ failure assessment (SOFA) score for group D and group P for a seven-day period.

The two groups showed similar SOFA scores during the study (Figure 2). No differences were found in coagulation disorders (platelet count), liver disorders (serum bilirubin), kidney disorders (serum creatinine) or central nervous system dysfunction (according to the Glasgow scale).

**Figure 3.**
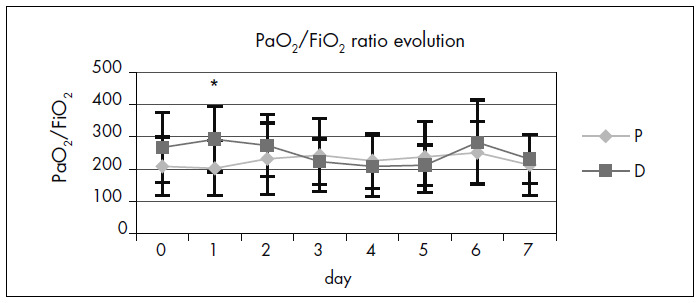
Significant improvement (*) in PaO^2^/FiO^2^ ratio during the first day in Group D (p = 0.041).

Over the first 24 hours after dexamethasone administration, the respiratory system showed an improved PaO^2^/FiO^2^ ratio (Mann-Whitney test; p = 0.041). However, this improvement did not persist throughout the study (Figure 3). The duration of mechanical ventilation was 3.4 ± 2.5 days for Group D and 4.0 ± 3.2 days for Group P (p = 0.22).

**Figure 4.**
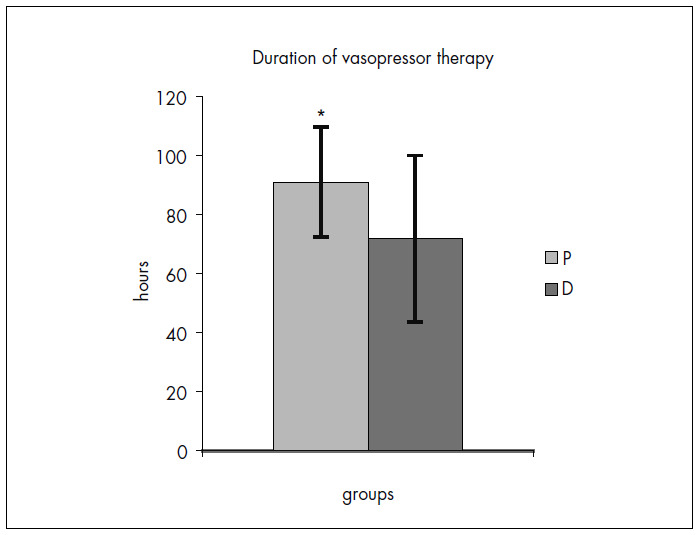
Duration in hours of vasopressor therapy for Group D and Group P (*p = 0.042).

The duration of vasopressor therapy was statistically different between the groups: 71.9 ± 28.2 hours per patient for Group D and 91.1 ± 18.6 hours for Group P (p = 0.042) (Figure 4).

**Figure 5.**
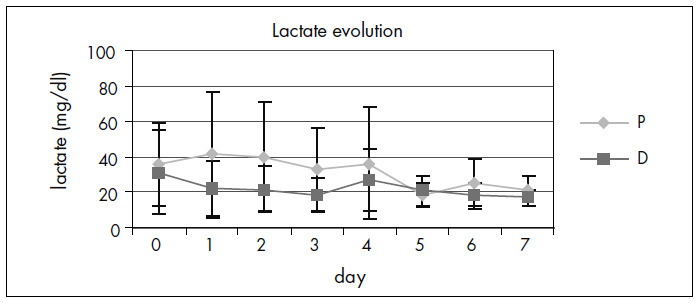
Evolution of lactate concentration (mg/dl) for Group D and Group P over a seven-day period.

The two groups were similar in relation to lactate assays during the seven-day period (Figure 5).

## DISCUSSION

Dexamethasone enhances the effects of vasopressor drugs and evaluation of the respiratory system showed improvements (better PaO^2^/FiO^2^ ratio) over the first day after its administration. Early treatment with dexamethasone reduced seven-day mortality among septic shock patients and showed a trend towards reduction of 28-day mortality.

Dexamethasone was chosen because of its potency and long-lasting action (36-48 hours) and its higher anti-inflammatory and lower mineralocorticoidal effects. In comparison with hydrocortisone, dexamethasone causes no changes in sodium reabsorption and does not interfere in the water balance, thus avoiding hypervolemia and sodium disturbances.^10^ We had already tested dexamethasone in SIRS patients with some improvements,^7^ so we decided to extend that study to septic patients in order to investigate its benefits and observe any possible adverse effects.

The pathophysiology of sepsis includes host inflammatory response, endothelial damage, increased coagulation with decreasing fibrinolysis, fibroproliferation and microclot formation and relative adrenal insufficiency.^17^ However, this systemic inflammatory response may lead to organ dysfunction instead of protecting and regulating homeostasis.^17^

Corticosteroids can improve the effects of vasopressor drugs, by reestablishing receptor sensitivity, with better effects using lower doses.^18^ The first explanation for the hemodynamic improvement seen in patients receiving corticosteroids was based on observations of the relative adrenal insufficiency that they might develop.^18-20^ In addition, some published reports have shown that patients without relative adrenal insufficiency could display better evolution following corticosteroid therapy.^1,21^ These reports may serve to support our results of early discontinuation of vasopressor therapy among patients receiving dexamethasone.

Currently, the recommendations for corticosteroids and sepsis are that this class of drugs should be used during refractory septic shock, but not during severe sepsis in the absence of shock or when only mild shock is observed.^6^ Whether or not sepsis is the systemic inflammatory response to infection, sepsis, severe sepsis and septic shock constitute different gradations in the continuum of a disease process. Moreover, the continuum of this process is correlated with increasing organ dysfunction and mortality. Early infusion of corticosteroids to block the process that began with an inflammatory reaction deserves to be tested.

The action of corticosteroids in septic patients can be explained by the relative adrenal insufficiency of these patients, but it seems to us that the principal mechanism of action of corticosteroids is based on their anti-inflammatory effect. Several studies have been using hydrocortisone following a corticotropin stimulation test.^20^ However, it needs to be asked whether the corticotropin stimulation test is really necessary. We have been using dexamethasone 0.2 mg/kg in SIRS patients and we have not observed any adverse effects at this dose. Therefore, even when including patients with adequate adrenal reserves, the use of corticosteroids at "physiological" doses will not lead to adverse effects like gastrointestinal hemorrhage or secondary infections.

An experimental study showed that corticosteroids decreased pulmonary edema and collagen formation.22 Another study demonstrated an improvement among patients with ARDS, following corticosteroid therapy, probably because of inhibition of pulmonary fibroproliferation.^5,23^ These previous studies support our observation that patients treated with dexamethasone displayed a better PaO^2^/FiO^2^ ratio on the first day after therapy. However, the use of corticosteroids for treating the early phase of acute lung injury (ALI) and ARDS has not been recommended (the recommendations include only the fibroproliferation phase).^24^ Even the patients in this study who received dexamethasone during the early exudative phase (days 1-5) of ALI/ARDS showed an improved PaO^2^/FiO^2^ ratio. The rationale may include the observation that the integrity of the epithelial barrier in resolving the alveolar edema appears to be a determining factor in the outcome for ARDS patients. Patients who can concentrate the protein in the edematous fluid during the first 12 hours of illness are more likely to recover than those who cannot. Finally, since the change in the PaO^2^/FiO^2^ ratio following the initial treatment for ARDS could pre-discriminate between survivors and non-survivors,^24^ the use of corticosteroids in the early phases of ALI/ARDS might be considered to be a reasonable measure.

## CONCLUSION

Dexamethasone enhanced the effects of vasopressor drugs and early treatment with dexamethasone reduced the seven-day mortality among septic shock patients.
